# Strengthening global biosecurity for synthetic nucleic acid technology: from sequence screening to risk-based governance in the AI era

**DOI:** 10.3389/fbioe.2026.1820001

**Published:** 2026-07-28

**Authors:** Sebunya Emmanuel Kato, Abigarl Ndudzo, Sarah Olivia Bayiga, Namugamba Brenda, Charity Serwaa, Erikan Baluku, Hazvinei Mang’anda, Joseph Jacob Peter, Kisuule Shamirah, Mweine Perez, Rosemond Nyatefe Tawiah, Simon Anguzu, Steven Malemia

**Affiliations:** SynBio4ALL Africa, Kampala, Uganda

**Keywords:** biosecurity governance, global biosecurity, risk governance, sequence screening, synthetic nucleic acid screening

## Abstract

Synthetic nucleic acid technology has expanded rapidly, reducing the cost, time, and technical barriers associated with DNA and RNA synthesis. Existing governance frameworks have focused mainly on sequence-based screening of synthesis orders and customer verification. Although these measures remain essential, they are no longer sufficient as standalone safeguards in a landscape shaped by AI-assisted biological design, globally distributed synthesis capacity, uneven regulatory implementation, and unclear liability across the design-synthesis-use pathway. Protein language models, generative design tools, and other AI-assisted approaches may expand the sequence design space in ways that are harder to assess through homology-based screening alone. This review examines the current biosecurity architecture for synthetic nucleic acid technology and identifies structural limitations in sequence-centered governance. We propose a systems-based, risk-informed framework organized around four interacting dimensions: design capability, access, governance strength, and liability and attribution clarity. The framework is diagnostic and semi-structured rather than quantitative, and is intended to complement existing screening standards and national regulatory approaches. Building on it, we present a four-tier governance escalation model that links dimension profiles to proportionate policy responses and assigns responsibilities to synthesis providers, research institutions, governments, funders, and international bodies. A worked case study illustrates how the framework can be applied to a plausible governance scenario with growing synthetic biology capacity and uneven oversight. The approach integrates equity and proportionality as governance constraints, supporting biosecurity strengthening without undermining responsible innovation.

## 1 Introduction

Over the past 2 decades, advances in synthetic nucleic acid technology have transformed biotechnology research and practice. Automation, high-throughput synthesis platforms, improved error-correction techniques, and large-scale oligonucleotide assembly methods have substantially reduced the cost, time, and technical barriers associated with designing and producing DNA constructs (National Academies of Sciences, Engineering, and Medicine, 2018). These capabilities have enabled progress across vaccine development, diagnostics, metabolic engineering, environmental biosensing, and synthetic genomics (National Academies of Sciences, Engineering, and Medicine, 2018).

As technical barriers declined, biosecurity concerns grew correspondingly. The possibility that gene synthesis services could be used to reconstruct known pathogens prompted the development of sequence screening mechanisms that compare orders against databases of regulated agents, select pathogens, and sequences of concern (National Academies of Sciences, Engineering, and Medicine, 2018; Diggans and Leproust, 2019). Industry-led standards, particularly those established through the International Gene Synthesis Consortium, introduced voluntary screening protocols and customer verification procedures (National Academies of Sciences, Engineering, and Medicine, 2018; Aspr, 2023). National governments supported these efforts through formal guidance, including documents issued by the U.S. Department of Health and Human Services (Aspr, 2023).

These frameworks were designed for a risk environment in which threats were primarily linked to known pathogenic sequences and synthesis was concentrated among a relatively small number of centralized commercial providers (Diggans and Leproust, 2019; Aspr, 2023). That environment has changed in ways that challenge the original assumptions. Computational design tools, including AI-assisted approaches, may expand the sequence design space in ways that are harder to evaluate using similarity-based screening alone (O’Brien and Nelson, 2020; Pauwels, 2023). Synthesis capacity is becoming more geographically distributed, and emerging benchtop synthesis platforms may complicate oversight when they fall outside conventional provider-based controls (Aspr, 2023; International Gene Synthesis Consortium, 2024). Regulatory implementation varies considerably across jurisdictions, while liability and attribution frameworks often remain unclear in cross-border misuse scenarios (van Baalen et al., 2023; Beal and Alexanian, 2025). Taken together, these pressures suggest that screening-centered governance, while still necessary, is no longer sufficient on its own.

Although existing work has examined synthetic biology risks, gene synthesis screening, AI-biotechnology convergence, and provider-level standards, these discussions are often treated as separate governance problems (National Academies of Sciences, Engineering, and Medicine, 2018; Diggans and Leproust, 2019; O’Brien and Nelson, 2020; Pauwels, 2023; Beal and Alexanian, 2025). This review contributes by integrating four interacting dimensions, namely, design capability, access, governance strength, and liability and attribution clarity, into a systems-based framework and operationalizing them through a four-tier governance escalation model. The aim is not to replace existing screening or customer verification approaches, but to provide a diagnostic structure that links risk conditions to proportionate policy responses across diverse regulatory contexts.

## 2 Current governance architecture

### 2.1 Sequence-based screening

Sequence-based screening is the foundation of current biosecurity oversight for synthetic nucleic acid providers. In practice, providers compare ordered sequences against curated databases of known pathogens, toxins, regulated agents, and sequences of concern, flagging matches above defined thresholds for further review (Diggans and Leproust, 2019; Aspr, 2023; International Gene Synthesis Consortium, 2024). Screening operates at both the nucleotide and protein levels. Nucleotide-level comparison detects direct or near-direct sequence similarity, while translated protein-level screening can identify similarity that remains relevant even when the underlying nucleotide sequence has been modified (Diggans and Leproust, 2019; Aspr, 2023). Customer verification complements sequence screening by assessing the legitimacy of the ordering institution, the identity of the customer, and the plausibility of the stated research purpose, reflecting that risk depends not only on what is ordered but also on who is ordering it and why (Diggans and Leproust, 2019; Aspr, 2023; International Gene Synthesis Consortium, 2024).

These measures have reduced the likelihood that known pathogenic sequences can be obtained from participating commercial providers without scrutiny. However, the effectiveness of similarity-based screening depends partly on the assumption that sequences of concern will retain detectable resemblance to entries in reference databases. This creates several limitations. Codon optimization and synonymous substitutions can reduce nucleotide-level similarity while preserving the encoded protein sequence or biological function (National Academies of Sciences, Engineering, and Medicine, 2018; Aspr, 2023). AI-assisted and other computational design approaches may further expand the sequence design space in ways that are difficult to evaluate using homology-based screening alone (O’Brien and Nelson, 2020; Pauwels, 2023; Wittmann et al., 2025). The growth of decentralized synthesis platforms, including benchtop devices, further challenges the assumption that screening will always occur through centralized commercial providers (Aspr, 2023; Gillum and Moritz, 2025). Differences in database coverage, threshold settings, and implementation standards across providers and jurisdictions add additional inconsistency (van Baalen et al., 2023; Beal and Alexanian, 2025).

In response to these limitations, researchers and developers have begun exploring screening approaches that move beyond direct sequence homology. Profile-based approaches, including hidden Markov models, can identify more remote functional relationships by detecting conserved sequence patterns across related protein families rather than relying only on close pairwise identity (Eddy, 1998; 2011). Structure-informed approaches use predicted or experimentally known protein structure to assess whether functional similarity may be conserved despite divergence at the primary sequence level (Jumper et al., 2021; Abramson et al., 2024). Embedding-based and machine-learning-assisted methods, including approaches derived from protein language models, represent sequences in high-dimensional feature spaces that may capture functional or evolutionary relationships not visible to conventional alignment alone (Rives et al., 2021; Lin et al., 2023; Wittmann et al., 2025). These methods are promising for strengthening functional screening, but their operational integration into routine provider screening remains limited and requires further validation (Wittmann et al., 2025).

It is important not to overstate what these emerging methods currently offer. Functional, structure-informed, and embedding-based screening approaches can complement conventional screening, but they do not eliminate the need for curated databases, expert review, customer verification, and governance oversight. Their value lies in extending coverage to parts of the design space that homology-based methods are less equipped to evaluate. Understanding where current screening methods are strongest and weakest is therefore essential for designing governance frameworks that are appropriately calibrated to the changing risk landscape. The main screening approaches, their strengths, and their limitations are summarized in Table 1.

**TABLE 1:** Comparison of screening approaches for synthetic nucleic acid biosecurity

Screening approach	Basis of detection	Key strength	Key limitation
Nucleotide-level alignment	Direct or near-direct nucleic acid sequence similarity	Sensitive for known sequences and close variants	Can be weakened by codon optimization or synonymous substitutions
Protein-level alignment	Similarity after translation into amino acid sequence	Detects functionally relevant similarity despite nucleotide changes	Still depends on reference database coverage and similarity thresholds
Profile-based screening	Conserved sequence patterns, motifs, or protein family profiles	Can detect more remote functional relationships	Requires well-curated profiles and expert interpretation
Structure-informed screening	Predicted or known three-dimensional protein structure	May identify conserved functional relationships despite sequence divergence	Computationally demanding and not yet routine in provider workflows
Embedding-based or ML-assisted screening	Learned sequence representations from large biological datasets	May capture non-obvious functional or evolutionary relationships	Requires validation, interpretability, and governance standards before operational use

### 2.2 National and international governance landscape

Governance of synthetic nucleic acid technology has developed unevenly across jurisdictions. This variation reflects differences in legal authority, regulatory capacity, biosafety culture, innovation strategy, export control systems, and the extent to which governments rely on voluntary industry standards. No binding global standard currently requires all nucleic acid synthesis providers to apply the same sequence screening and customer verification procedures. As a result, the level of oversight applied to a synthesis order can depend less on the risk profile of the order itself than on the provider, jurisdiction, and institutional context in which the order is placed. A comparison of the main governance approaches for synthetic nucleic acid oversight is provided in Table 2.

**TABLE 2:** Comparative governance approaches for synthetic nucleic acid oversight.

Jurisdiction or body	Main governance approach	Screening status	Key strength	Key limitation
United States	Federal guidance, funding-linked expectations, industry engagement	Encouraged, with stronger expectations emerging	Adaptable and linked to provider practice	Uneven implementation and benchtop-device gaps
European Union	Dual-use controls, biosafety obligations, precautionary regulation	No fully harmonized EU-wide mandatory screening framework yet	Strong regulatory tradition	Fragmented synthesis-specific implementation
United Kingdom	Broader biological security strategy	Developing	Integrated national biosecurity framing	Synthesis-specific mechanisms still evolving
Selected Asian jurisdictions	Mixed biosafety, biotechnology, export control, and institutional oversight systems	Uneven and jurisdiction-specific	Growing biotechnology capacity and policy attention	Publicly documented synthesis-specific screening rules remain inconsistent
IGSC	Voluntary industry screening standards	Applies to member companies	Operationally detailed provider guidance	Limited to participating providers
Biological weapons convention	International prohibition of biological weapons	No synthesis-specific screening mechanism	Broad international norm against biological weapons	Lacks operational rules for screening and provider obligations

In the United States, federal guidance has encouraged providers and users of synthetic nucleic acids to apply sequence screening, customer verification, and recordkeeping practices (Aspr, 2023). More recent federal action has moved toward stronger expectations for screening, including in relation to federally funded research purchases and AI-enabled biological design risks (OSTP Framework for Nucleic Acid Synthesis Screening of for NASS., 2024). However, implementation remains uneven across the provider ecosystem, particularly where synthesis capacity falls outside conventional commercial provider relationships, including emerging benchtop synthesis platforms (Aspr, 2023; Gillum and Moritz, 2025). The U.S. approach therefore illustrates both the strengths and limits of guidance-based governance: it can adapt quickly, but its effectiveness depends on uptake, incentives, and implementation capacity.

In Europe, synthetic nucleic acid governance is shaped by dual-use export controls, biosafety obligations, and broader precautionary regulatory traditions. However, a unified mandatory framework for sequence-level screening of gene synthesis orders has not yet been fully established across the European Union (European Commission, 2025). The United Kingdom has addressed biological security through broader biosecurity and biological security strategies, but synthesis-specific screening governance remains part of an evolving policy landscape (UK Government, 2023). These examples show that strong general biosecurity systems do not automatically translate into harmonized oversight of individual synthesis transactions.

Asian governance landscapes also differ substantially across jurisdictions and should not be treated as a single model. China, Japan, India, and Singapore differ in their biotechnology capacity, regulatory traditions, biosafety institutions, and innovation priorities. Some jurisdictions have developed broad biosafety or biotechnology governance frameworks, while others rely more heavily on institutional oversight, export control systems, or laboratory biosafety rules. However, publicly documented, synthesis-specific screening requirements remain uneven across the region (van Baalen et al., 2023). This variation matters because the practical effectiveness of screening depends not only on national policy commitments, but also on institutional capacity, provider participation, and enforceable implementation mechanisms.

Industry-led governance has helped to reduce some of these gaps. The International Gene Synthesis Consortium has developed voluntary screening standards that include sequence screening and customer verification for member companies (Diggans and Leproust, 2019; International Gene Synthesis Consortium, 2024). These standards are among the most operationally detailed mechanisms currently available to providers. Their limitation is coverage: companies outside participating networks are not bound by the same procedures, and voluntary standards do not create uniform expectations across jurisdictions (Beal and Alexanian, 2025).

At the international level, the Biological Weapons Convention establishes broad prohibitions against the development, production, acquisition, and transfer of biological weapons. However, it does not provide detailed operational rules for gene synthesis screening, provider obligations, benchtop synthesis devices, or AI-assisted biological design (Sanz et al., 2022; Alexanian, 2024). This creates a gap between high-level biosecurity norms and the practical governance of synthetic nucleic acid transactions. International discussions have increasingly recognized synthetic biology as relevant to biological weapons governance, but binding technical standards for synthesis screening have not yet emerged.

The policy lesson is that harmonization should not mean identical regulation everywhere. A shared baseline for sequence screening, customer verification, recordkeeping, and escalation of suspicious orders would reduce inconsistent oversight across providers and jurisdictions. At the same time, implementation must remain sensitive to national capacity and innovation equity. Requirements that are technically or financially difficult for lower-resource settings to implement may widen participation gaps unless they are paired with training, shared screening infrastructure, technical assistance, and clear pathways for responsible access.

## 3 Structural pressures in the contemporary landscape

### 3.1 Expanding design capability

The ability to design biological sequences has expanded with advances in computational biology, machine learning, and generative protein design. This matters for biosecurity because risk can emerge before synthesis, at the design stage, when sequences are generated, optimized, or modified before any physical material is produced. Conventional sequence-screening systems were developed largely around the synthesis transaction, where providers assess whether an ordered sequence resembles known pathogens, toxins, regulated agents, or sequences of concern (Diggans and Leproust, 2019; Aspr, 2023).

AI-assisted design tools challenge this checkpoint model by expanding the range of sequence candidates that can be generated or optimized. Protein language models learn statistical representations from large biological sequence datasets and can support prediction, representation, or generation tasks relevant to protein engineering (Rives et al., 2021; Lin et al., 2023). Diffusion-based protein design tools can generate protein structures or sequences guided toward specified structural or functional objectives (Watson et al., 2023). These tools have important beneficial applications in medicine, biotechnology, and basic science, but they also complicate screening systems that rely mainly on similarity to known sequences. Recent work has shown that generative protein design tools can produce redesigned variants of proteins of concern that are more difficult for existing nucleic acid screening systems to detect, although improved screening patches can substantially reduce this vulnerability (Wittmann et al., 2025). The governance issue, therefore, is not that AI-generated sequences are automatically dangerous, but that a larger and less familiar design space weakens the assumption that risk can be managed only by comparing orders against known sequence databases.

This distinction also separates design-stage risk from synthesis-stage risk. A computationally generated sequence does not become a biological material until it is synthesized, assembled, and used. However, decisions made at the design stage can shape what later enters synthesis workflows. This suggests the need for layered governance, including responsible access to high-capability design tools, developer-side screening or safety evaluation where appropriate, provider-level synthesis screening, and institutional oversight for users working with potentially concerning designs (Pauwels, 2023; OSTP Framework for Nucleic Acid Synthesis Screening of for NASS., 2024).

### 3.2 Expanding access

Access to synthetic nucleic acid technology has also broadened. Commercial gene synthesis providers now serve international customers, while biofoundries and automated design-build-test workflows have expanded the speed and scale of biological engineering (National Academies of Sciences, Engineering, and Medicine, 2018; Diggans and Leproust, 2019). Digital collaboration platforms allow sequence designs, protocols, and analytical tools to move across borders more easily than physical materials, separating the design, ordering, synthesis, and use stages across different actors and jurisdictions.

Benchtop nucleic acid synthesis platforms add another access-related pressure. Conventional governance relies heavily on commercial providers as screening checkpoints, but benchtop devices can shift some synthesis activity into individual laboratories or institutional settings. U.S. screening guidance and the 2024 federal screening framework explicitly recognize this issue by including benchtop nucleic acid synthesis equipment within the scope of screening expectations for federally funded research purchases (Aspr, 2023; OSTP Framework for Nucleic Acid Synthesis Screening of for NASS., 2024). This does not mean provider-level screening is obsolete. It means governance must account for a more distributed synthesis environment in which screening responsibilities may need to extend beyond traditional commercial order review.

### 3.3 Liability and attribution gaps

Responsibility for synthetic nucleic acid misuse can be distributed across multiple actors, including design-tool developers, sequence designers, synthesis providers, research institutions, funders, and end users. These actors may operate under different legal systems, particularly when design, synthesis, shipment, and use occur across borders. This creates difficulty in assigning responsibility when suspicious orders, unsafe designs, or harmful outcomes arise (Pauwels, 2023; Beal and Alexanian, 2025).

Provider-level standards can clarify obligations at one important point in the pathway, especially where providers are required or strongly incentivized to screen sequences, verify customers, retain records, and escalate suspicious orders (Aspr, 2023). However, provider obligations do not fully resolve upstream and downstream accountability. Tool developers may influence what designs are generated, institutions may determine whether users are trained and supervised, and national authorities may differ in how they regulate biosecurity, export controls, data retention, and liability. Attribution is further complicated when design decisions are not recorded, when synthesis occurs through multiple intermediaries, or when activity crosses jurisdictions with different enforcement capacities.

### 3.4 Governance fragmentation

These pressures are reinforced by governance fragmentation. Existing mechanisms, including voluntary industry standards, national guidance, export controls, biosafety rules, and the Biological Weapons Convention, each address part of the biosecurity problem but do not form a fully integrated governance system (International Gene Synthesis Consortium, 2024; Wittmann et al., 2025). The result is inconsistent oversight across providers and jurisdictions. A synthesis order may receive different levels of review depending on whether the provider follows IGSC standards, whether national guidance applies, whether the customer is subject to funding-linked requirements, and whether local institutions have the capacity to enforce biosafety and biosecurity rules.

Fragmentation also has an equity dimension. Higher-resource settings are generally better positioned to implement advanced screening, audit systems, legal oversight, and technical training. Lower-resource settings may face capacity constraints even when there is political will to strengthen biosecurity. A credible governance model should therefore avoid treating harmonization as a purely legal or technical exercise. It should pair shared baseline expectations with capacity-building, technical assistance, and proportional requirements that do not unnecessarily restrict responsible biotechnology development.

## 4 Limitations of sequence-centered governance

Sequence-based screening, customer verification, and provider-level oversight form the operational core of current biosecurity governance for synthetic nucleic acid technology. These measures have reduced the likelihood that known pathogenic sequences can be obtained from participating commercial providers without scrutiny, and they remain essential components of any credible governance system (Diggans and Leproust, 2019; International Gene Synthesis Consortium, 2024). The issue is therefore not whether screening should be retained, but whether sequence-centered governance is sufficient on its own for the current risk landscape. Its limitations are structural as well as technical.

First, sequence screening is anchored to known sequence similarity. Databases of sequences of concern are necessarily based on existing knowledge, which makes screening strongest when ordered sequences closely resemble known pathogens, toxins, regulated agents, or other documented sequences of concern (National Academies of Sciences, Engineering, and Medicine, 2018). Sensitivity may decline as sequences diverge from known references, particularly where codon optimization, synonymous substitution, or computational design approaches preserve biological function while reducing direct similarity (O’Brien and Nelson, 2020). This is not a failure of screening as a concept, but a boundary condition of similarity-based methods that governance systems need to recognize explicitly.

Second, provider-level oversight captures only one point in a longer pathway from design to use. The design stage, including AI-assisted optimization, generative protein design, and other computational workflows, can occur before any synthesis order is placed (Pauwels, 2023). Digital sequences may also be shared, modified, and transferred across borders before they enter a synthesis workflow. In addition, benchtop synthesis platforms can shift some synthesis activity into laboratories or institutional settings rather than centralized commercial providers (Aspr, 2023; Gillum and Moritz, 2025). A governance architecture that concentrates safeguards at the provider transaction therefore remains exposed to activity that precedes, bypasses, or falls outside conventional commercial screening.

Third, implementation remains fragmented. Voluntary standards applied by International Gene Synthesis Consortium members do not bind non-member providers, and national approaches to screening, customer verification, recordkeeping, and enforcement differ across jurisdictions (International Gene Synthesis Consortium, 2024). The practical consequence is that similar synthesis transactions may receive different levels of review depending on the provider, country, customer type, and applicable policy framework. This inconsistency weakens system-wide governance because oversight is shaped by institutional location and provider participation as much as by the risk profile of the order.

Fourth, sequence-centered governance is weakly connected to liability and attribution. Screening can document whether an order was reviewed, but it does not by itself establish clear responsibility across the wider actor chain involved in design, synthesis, distribution, and use. Responsibility may be distributed among tool developers, sequence designers, synthesis providers, research institutions, funders, and end users, often across different legal systems (Beal and Alexanian, 2025). When misuse occurs, existing mechanisms for assigning responsibility across this chain remain uneven and underdeveloped. These structural limitations and their governance implications are summarized in Table 3.

**TABLE 3:** Structural limitations of sequence-centered governance and their implications.

Structural limitation	Governance implication	Corresponding framework dimension
Dependence on known sequence similarity	Need to account for design-stage risks and emerging functional screening methods	Design capability
Focus on provider-level checkpoints	Need for safeguards upstream and downstream of the synthesis transaction	Access
Fragmented implementation across providers and jurisdictions	Need for harmonized baselines and stronger coordination	Governance strength
Weak integration with responsibility and attribution mechanisms	Need for clearer accountability across the design-synthesis-use pathway	Liability and attribution clarity

Taken together, these limitations point toward a governance approach that retains screening as a core safeguard while embedding it within a broader, adaptive framework. Addressing the known-sequence boundary condition requires attention to design capability and emerging functional screening methods. Addressing the provider-checkpoint limitation requires governance mechanisms that extend upstream and downstream of the synthesis transaction. Addressing fragmented implementation requires stronger baseline coordination across providers and jurisdictions. Addressing liability and attribution gaps requires clearer responsibility across the actor chain. The four-dimension framework developed in the next section is designed to integrate these requirements into a single analytical structure.

## 5 A systems-based risk framework

The limitations identified above cannot be addressed by improving any single governance instrument in isolation. Better screening tools can strengthen detection, but they do not resolve provider coverage gaps, uneven implementation, or unclear responsibility across the design-synthesis-use pathway. Similarly, harmonized standards can reduce fragmentation, but they do not by themselves address design-stage risks created by expanding computational capability. A broader framework is therefore needed to assess how different risk conditions interact.

We propose a semi-structured diagnostic framework organized around four interacting dimensions: design capability, access, governance strength, and liability and attribution clarity. The framework is not a validated quantitative scoring model. The definitions, indicators, assessment sources, and governance relevance of these dimensions are presented in Table 4. Rather, it is intended to help regulators, synthesis providers, research institutions, and international bodies characterize a governance context, identify where risk is concentrated, and select proportionate responses. It complements existing mechanisms such as sequence screening, customer verification, provider standards, and national biosafety regulations rather than replacing them (Diggans and Leproust, 2019; Aspr, 2023; Beal and Alexanian, 2025).

**TABLE 4:** Operationalizing the four-dimension risk framework.

Dimension	Definition	Possible indicators or proxies	Assessment sources	Governance relevance
Design capability	Ability to design novel or modified biological sequences using computational, experimental, or institutional resources	Synthetic biology research groups; biofoundry presence; computational biology expertise; access to generative design tools; publication or patent activity	Publication databases; biofoundry registries; national science and technology reports; institutional capability assessments	High capability may expand the range of sequences entering synthesis workflows and may require design-stage governance
Access	Ease with which actors can obtain synthesized nucleic acids through monitored or unmonitored channels	Number and reach of synthesis providers; online ordering; IGSC participation; customer verification practices; benchtop synthesis capacity	Provider directories; IGSC membership records; national guidance documents; market and infrastructure reports	Broad unmonitored access increases the number of actors able to convert sequence designs into physical material
Governance strength	Robustness of legal, regulatory, and institutional oversight mechanisms	Mandatory or voluntary screening requirements; competent authority; audit or sanction mechanisms; institutional biosafety and biosecurity committees; participation in international biosecurity instruments	National legislation; regulatory guidance; treaty reports; biosecurity governance analyses	Strong governance improves implementation, coordination, and enforcement of safeguards
Liability and attribution clarity	Ability to assign responsibility and investigate misuse across the design-synthesis-use pathway	Recordkeeping duties; traceability or provenance systems; contractual screening obligations; institutional responsibilities; cross-border cooperation mechanisms	Provider policies; national law; legal analyses; forensic and traceability literature	Clear responsibility supports deterrence, investigation, and accountability

Design capability refers to the extent to which actors can use computational, experimental, and institutional resources to design novel or modified biological sequences. Relevant indicators include the presence of synthetic biology research groups, biofoundries, computational biology expertise, protein engineering capacity, access to generative design tools, and publication or patent activity in synthetic genomics and protein design. This dimension matters because high design capability can expand the range of sequences that may enter synthesis workflows, including sequences that are harder to evaluate using similarity-based screening alone (National Academies of Sciences, Engineering, and Medicine, 2018).

Access refers to the ease with which actors can obtain synthesized nucleic acids through commercial providers, distributed services, benchtop platforms, or collaborative arrangements. Relevant indicators include the number and geographic reach of synthesis providers, the availability of online ordering, participation in screening networks such as the International Gene Synthesis Consortium, the use of customer verification procedures, and the presence of benchtop synthesis capacity. Access is not itself a biosecurity problem, since broad access is essential for beneficial research. The concern is unmonitored or weakly governed access that allows sequence specifications to be converted into physical material without adequate screening or accountability (International Gene Synthesis Consortium, 2024).

Governance strength refers to the robustness of the legal, regulatory, and institutional mechanisms used to oversee synthetic nucleic acid technology. Relevant indicators include whether screening requirements are voluntary or mandatory, whether competent authorities have clear legal mandates, whether enforcement mechanisms such as audits or sanctions exist, whether institutional biosafety and biosecurity committees have relevant scope, and whether the jurisdiction participates in international biosecurity instruments. Governance strength matters because formal rules are only effective when they are implemented, enforceable, and coordinated across relevant actors (Alexanian, 2024).

Liability and attribution clarity refers to the extent to which responsibility can be assigned across the actor chain involved in design, synthesis, distribution, and use. Relevant indicators include provider recordkeeping requirements, traceability or provenance mechanisms, contractual duties for screening and reporting, legal responsibilities for institutions and end users, and cross-border cooperation mechanisms for biosecurity incidents. This dimension matters because unclear responsibility weakens deterrence and makes it harder to investigate or respond to suspected misuse, especially when design, synthesis, and use occur across different jurisdictions (Pauwels, 2023).

The framework treats risk as interaction-based rather than purely additive. High design capability and broad access increase exposure because more actors are able to specify and obtain synthetic sequences. Strong governance and clear liability can reduce that exposure by creating screening obligations, review procedures, recordkeeping, escalation pathways, and accountability mechanisms. Conversely, high capability and broad access combined with weak governance and unclear liability create the most concerning risk conditions. This does not mean that each dimension should be converted into a precise numerical score. In most settings, the available evidence is qualitative or semi-quantitative. The framework is therefore best used as a structured assessment tool that supports tier assignment in the escalation model developed in the next section.

Equity and proportionality should guide how the framework is applied. A uniform global requirement that is technically or financially unrealistic in lower-resource settings may reduce participation in responsible biotechnology without improving security. The purpose of the framework is therefore not to impose identical controls everywhere, but to identify where governance support, technical assistance, shared screening infrastructure, or stronger accountability mechanisms are most needed. This allows biosecurity strengthening to be linked to responsible innovation rather than treated as a restriction on it.

## 6 Risk-tiered governance escalation

### 6.1 From framework to governance response

The four-dimension framework provides a diagnostic structure for assessing synthetic nucleic acid biosecurity risk. The tier model translates that diagnosis into proportionate governance responses. It links combinations of design capability, access, governance strength, and liability and attribution clarity to escalating levels of oversight. The entry criteria, illustrative scenarios, governance responses, and lead responsibilities for each tier are summarized in Table 5.

**TABLE 5:** Four-tier governance escalation model.

Tier	Entry criteria	Illustrative scenario	Main governance response	Lead responsibilities
Tier 1: Controlled environment	Moderate or high design capability; access mainly through screened providers; strong governance; clear responsibility pathways	Research university using screened providers under institutional biosafety oversight and national screening guidance	Maintain baseline screening, customer verification, recordkeeping, institutional review, and periodic implementation review	Providers: Screen orders and verify customers. Institutions: Train researchers and review relevant projects. Governments: Update guidance and monitor implementation. International bodies: Support information sharing
Tier 2: Transitional risk	Expanding capability or access; partial screening adoption; uneven institutional oversight; incomplete liability rules	Growing synthetic biology sector with some voluntary screening participation and laboratory biosafety rules, but no synthesis-specific requirements	Strengthen provider participation in screening standards; expand institutional oversight; introduce training; clarify recordkeeping and reporting expectations	Providers: Adopt recognized standards. Institutions: Expand biosafety committee scope. Governments: Clarify screening expectations. Funders: Require oversight evidence. International bodies: provide technical assistance
Tier 3: Elevated risk	Two or more dimensions significantly deficient, or one severe gap; high capability or access with weak screening, enforcement, or liability	Biofoundry and computational design capacity with inconsistent provider screening and no synthesis-specific liability or reporting framework	Introduce mandatory screening, provider registration or licensing, audit mechanisms, suspicious-order reporting, institutional review for higher-concern designs, and cross-border coordination	Providers: Apply enhanced screening and maintain audit-ready records. Institutions: Maintain traceability. Governments: Enforce requirements. Funders: Require provider disclosure. International bodies: Support shared standards
Tier 4: High-risk ecosystem	High or rapidly expanding design capability; broad unmonitored access; weak or absent governance; unclear or unenforceable liability	Accessible AI-assisted design tools combined with unscreened synthesis channels, weak domestic regulation, and limited institutional biosafety infrastructure	Prioritize international engagement, minimum screening baselines, capacity-building, provider obligations, institutional biosafety infrastructure, and clearer accountability rules	Providers: Screen regardless of customer jurisdiction. Governments: Clarify obligations and use procurement, funding, or export-control tools where appropriate. International bodies: Coordinate assistance and shared expectations. Funders: Pair conditionality with capacity investment

The model is semi-structured rather than algorithmic. Tier assignment requires judgement using the indicators described in Section 5, and the appropriate response will vary across institutional and national contexts. The model should therefore not be treated as a validated quantitative scoring system. Its purpose is to support structured decision-making, clarify actor responsibilities, and avoid both under-regulation in high-risk contexts and unnecessary restriction in lower-risk settings.

Movement from Tier 1 to Tier 4 reflects increasing net risk. Risk increases where design capability and access are high or rapidly expanding, while governance strength and liability clarity remain weak. Risk decreases where screening, customer verification, institutional oversight, recordkeeping, enforcement, and accountability mechanisms are strong. A context may move to a higher tier because of a severe weakness in one dimension, even if other dimensions are moderate.

### 6.2 Tier 1: controlled environment

Tier 1 describes contexts where synthetic nucleic acid activity occurs within strong governance systems. Design capability may be moderate or high, but access to synthesis is predominantly routed through providers that apply sequence screening, customer verification, recordkeeping, and escalation procedures. Institutional biosafety and biosecurity oversight is functioning, and liability or responsibility pathways are reasonably clear.

An example would be a research university in a jurisdiction with strong screening expectations, active institutional biosafety review, researcher training, and routine use of providers that follow recognized screening standards. In this setting, the appropriate response is not additional restriction, but maintenance of baseline safeguards. Providers should apply standard screening and customer verification procedures. Institutions should maintain biosafety and biosecurity review for relevant projects. Governments should update guidance, monitor implementation, and ensure that recordkeeping and escalation pathways remain functional. International bodies can support database updating and information sharing on emerging screening practices.

### 6.3 Tier 2: transitional risk

Tier 2 describes contexts where capability or access is expanding faster than governance implementation, but where basic safeguards are present or feasible. This may include settings with growing synthetic biology research, partial provider participation in voluntary screening schemes, uneven institutional oversight, or liability frameworks that exist only through general biosafety or research governance rules.

An example would be a middle-income country with an expanding synthetic biology sector, some domestic or regional synthesis access, partial use of voluntary screening standards, and laboratory biosafety rules that do not yet include synthesis-specific requirements. Appropriate responses include strengthening provider participation in recognized screening standards, expanding institutional biosafety committee scope to include synthetic nucleic acid oversight, introducing researcher biosecurity training, and developing synthesis-specific recordkeeping and reporting expectations. Governments should clarify whether screening and customer verification are voluntary or mandatory. Funders can require evidence of institutional oversight for relevant projects. International bodies and higher-capacity partners should provide technical assistance, access to screening resources, and training rather than imposing unsupported compliance burdens.

### 6.4 Tier 3: elevated risk

Tier 3 describes contexts where two or more dimensions are significantly deficient, or where one dimension presents a severe governance gap. Typical Tier 3 conditions include high or growing design capability, broad synthesis access, weak or unenforced screening expectations, limited provider participation in recognized standards, and unclear liability for cross-border synthesis or use.

An example would be a jurisdiction with active biofoundry infrastructure and use of advanced computational design tools, but with voluntary screening that is not consistently implemented, several synthesis providers outside recognized screening networks, and no synthesis-specific liability or reporting framework. In this setting, governance should move beyond baseline guidance. Governments should consider mandatory screening requirements, provider registration or licensing, audit mechanisms, and reporting obligations for suspicious orders. Providers should apply nucleotide-level and protein-level screening, customer verification, recordkeeping, and escalation procedures. Research institutions should introduce internal review for projects involving *de novo* sequence design or higher-concern constructs, and should maintain traceability records for synthetic constructs. Funders can require disclosure of synthesis providers and screening procedures. International bodies can support cross-border coordination, shared technical standards, and capacity-building for regulators.

### 6.5 Tier 4: high-risk ecosystem

Tier 4 describes contexts or actor configurations in which high or rapidly expanding design capability combines with broad unmonitored access, weak or absent governance, and unclear or unenforceable liability. Tier 4 need not describe an entire country. It may describe a specific ecosystem, network, or pathway in which AI-assisted design tools, synthesis access, and weak oversight intersect across jurisdictions.

An example would be a setting where powerful design tools are widely accessible without institutional oversight, synthesis services can be obtained from providers that do not apply systematic screening or customer verification, domestic regulation does not address synthetic nucleic acid oversight, and institutional biosafety infrastructure is absent or very limited. In such conditions, unilateral restrictions alone are unlikely to create durable security. Priority responses should include international engagement, technical assistance, development of minimum screening and customer verification baselines, support for institutional biosafety infrastructure, and clearer responsibility rules across the design-synthesis-use pathway. Providers should apply screening and customer verification regardless of customer jurisdiction. Governments should clarify provider obligations and, where appropriate, use export control, procurement, or funding mechanisms to reduce unscreened access. International bodies should support capacity-building, coordination through existing biosecurity forums, and development of shared expectations for synthesis governance.

Equity is especially important in Tier 4. A high-risk designation should not be used simply to exclude lower-resource settings from synthetic biology. The goal should be risk reduction through capacity development, not permanent restriction. Movement from Tier 4 toward Tier 3 or Tier 2 should be supported through training, shared screening infrastructure, institutional oversight, and legal or regulatory assistance.

This Table 6 illustrates how combinations of design capability and access interact with governance strength and liability clarity to shape the overall governance tier. The vertical axis represents the level of design capability and access, ranging from low or limited to high or broad. The horizontal axis represents governance strength and liability clarity, ranging from strong or clear to weak or unclear. Tier assignment is indicative rather than purely mechanical, and reflects the interaction-based logic of the framework.

**TABLE 6:** Interaction logic of the four-tier escalation model.

Design capability and access	Strong governance and clear liability	Weak governance and unclear liability
High design capability and broad access	Tier 1 to tier 2 managed high-capability environment. High capability and access are present, but risks are moderated by screening, institutional oversight, enforceable rules, and clear accountability pathways	Tier 3 to tier 4 elevated or high-risk ecosystem. High capability and broad access combine with weak oversight and unclear responsibility, increasing the likelihood of unmonitored or unaccountable misuse
Low design capability and limited access	Tier 1 Controlled lower-risk environment. Lower capability and limited access operate within a strong governance setting, resulting in the lowest overall risk profile	Tier 2 to tier 3 structural governance gap requiring capacity-building. Capability and access are lower, but weak governance and unclear liability create vulnerabilities that may justify targeted governance support and institutional strengthening

## 7 Worked case study

### 7.1 Purpose and framing

This case study illustrates how the four-dimension framework and four-tier escalation model can be applied in practice. The example is a composite scenario based on a lower-resource or middle-income setting with growing synthetic biology capacity. It is not an assessment of a specific country and should not be read as a ranking of any jurisdiction. The purpose is to show how the framework can identify governance gaps, assign a risk tier, and guide proportionate responses without treating lower-resource settings as inherently risky.

### 7.2 Case profile

The case involves a setting in which synthetic biology research is expanding through universities, regional research centres, and international collaborations. Researchers have access to commercial gene synthesis services, open-access computational biology tools, and some automated design-build-test infrastructure. A small number of domestic or regional synthesis providers may be available, while many orders are still placed through international providers. Institutional biosafety systems exist in some research centres, but their scope is focused mainly on laboratory biosafety rather than upstream synthesis oversight or AI-assisted sequence design.

The national governance framework includes general biosafety rules and participation in international biosecurity norms, but it does not yet include synthesis-specific requirements for sequence screening, customer verification, provider registration, or suspicious-order reporting. Liability and attribution mechanisms are also limited. Responsibility for misuse is not clearly assigned across designers, institutions, synthesis providers, and end users, and recordkeeping practices are inconsistent.

### 7.3 Dimension-by-dimension assessment

Design capability is moderate and growing. The presence of trained researchers, international collaborations, computational design tools, and emerging design-build-test infrastructure means that actors can increasingly specify or modify biological sequences. However, advanced generative design expertise is concentrated in a limited number of groups, and access to proprietary platforms remains constrained by cost and infrastructure.

Access is moderate to high, with uneven oversight. Researchers can obtain synthesized nucleic acids through international providers and, in some cases, domestic or regional suppliers. Some providers may follow recognized screening standards, while others may not. Where benchtop synthesis platforms or informal ordering pathways exist, synthesis activity may fall outside provider-based screening systems.

Governance strength is weak to moderate. General biosafety rules and institutional oversight structures provide a foundation, but they do not fully address synthesis-specific risks. The absence of clear requirements for provider screening, customer verification, recordkeeping, and suspicious-order escalation creates implementation gaps. Institutional biosafety committees may also vary in their capacity to review projects involving *de novo* sequence design or AI-assisted design tools.

Liability and attribution clarity is low. Existing legal mechanisms may address general biosafety violations or research misconduct, but they do not clearly assign responsibility across the full design-synthesis-use pathway. If a concerning sequence is designed in one jurisdiction, synthesized through a provider in another, and used in a third, responsibility may be difficult to establish. Weak recordkeeping and limited traceability further complicate investigation and accountability. The dimension-by-dimension assessment, risk implications, recommended responses, and resulting tier assignment are summarized in Table 7.

**TABLE 7:** Application of the framework to the worked case study.

Framework dimension	Case condition	Risk implication	Recommended response
Design capability	Moderate and growing; trained researchers, computational tools, international collaborations, and emerging design-build-test infrastructure	Increasing ability to specify or modify biological sequences, including through computational design	Expand institutional review to include synthetic nucleic acid research and AI-assisted sequence design
Access	Moderate to high; use of international providers, possible domestic or regional suppliers, and uneven provider screening	Some synthesis activity may occur outside systematic screening and customer verification	Promote recognized screening standards, provider registration, customer verification, and shared screening support
Governance strength	Weak to moderate; general biosafety rules exist but synthesis-specific oversight is limited	Screening, recordkeeping, and suspicious-order escalation may be inconsistently applied	Extend biosafety governance to include synthesis screening, provider obligations, and escalation procedures
Liability and attribution clarity	Low; responsibility across designers, providers, institutions, and users is not clearly assigned	Investigation and accountability may be difficult, especially in cross-border transactions	Establish recordkeeping duties, clarify actor responsibilities, and develop cross-border information-sharing mechanisms
Tier assignment	Tier 3, elevated risk	Growing capability and access combined with weak governance and low liability clarity	Prioritize phased governance strengthening, capacity-building, and support for responsible innovation

### 7.4 Tier assignment

This case maps most closely to Tier 3, elevated risk. The rationale is that design capability and access are growing, while governance strength and liability clarity remain insufficiently developed. The case does not automatically fall into Tier 4 because capability and access are not assumed to be fully mature or broadly unmonitored across all pathways. However, the absence of synthesis-specific governance and clear accountability creates conditions that could move the setting toward Tier 4 if access and design capability continue to expand without parallel governance development.

### 7.5 Recommended governance responses

For government authorities, the priority is to extend existing biosafety governance to include synthetic nucleic acid oversight. Initial measures could include provider registration or notification, adoption of minimum screening and customer verification expectations, recordkeeping requirements, and a clear escalation pathway for suspicious orders. These measures could be introduced through a phased approach, beginning with guidance and capacity-building before moving toward mandatory requirements where feasible.

For synthesis providers, the priority is to adopt recognized screening practices, including nucleotide-level and protein-level screening, customer verification, recordkeeping, and escalation procedures. Where local providers lack technical capacity, shared screening services, regional consortia, or partnerships with established screening networks could provide an interim pathway.

For research institutions, institutional biosafety committees should expand their scope to include synthetic nucleic acid research, computational sequence design, and projects involving higher-concern constructs. Researcher training should include biosecurity responsibilities, responsible ordering practices, and procedures for escalating uncertain cases.

For funders and international partners, the priority is to pair oversight expectations with capacity-building. Funding conditions can require evidence of institutional biosafety review and responsible synthesis procurement, but these requirements should be accompanied by training, access to screening resources, support for regulatory development, and technical assistance for local providers.

This case illustrates why the framework is intended to support proportionate governance rather than blanket restriction. The appropriate response is not to limit responsible synthetic biology development in lower-resource settings, but to strengthen governance capacity alongside research capacity. In this way, the tier model can support both biosecurity and equitable participation in biotechnology innovation.

## 8 Responsible innovation and equity as governance safeguards

The framework proposed in this review treats equity and responsible innovation as constraints on how biosecurity governance should be applied. A risk-based model should not impose identical requirements across all settings regardless of capability, access, governance capacity, or liability clarity. Such an approach could overburden lower-capacity institutions while failing to address higher-risk configurations in better-resourced settings. Proportionality is therefore not only an ethical principle, but also a practical requirement for effective governance.

In this context, proportionality means matching safeguards to the actual risk conditions identified through the four-dimension framework. A lower-resource setting with limited synthesis access and emerging design capability presents a different governance problem from a high-capability setting with broad access and weak accountability mechanisms. Treating these contexts as equivalent may overstate risk in one setting and understate it in another. The tier model is intended to avoid this problem by linking governance responses to the interaction of design capability, access, governance strength, and liability clarity.

Several safeguards can help align biosecurity with responsible innovation. Phased implementation allows jurisdictions and institutions to move from guidance and voluntary standards toward stronger requirements as technical and regulatory capacity develops. Shared screening infrastructure, regional provider consortia, and supported access to screening resources can reduce the burden on smaller providers and lower-resource institutions. Training for researchers, institutional biosafety committees, and regulators can strengthen compliance without excluding responsible actors from synthetic biology research. Funders can also support this process by pairing biosecurity expectations with resources for implementation rather than treating compliance as an unsupported precondition.

Responsible access to computational design tools is also part of this balance. As AI-assisted biological design tools become more widely available, governance should focus not only on restricting access, but also on supporting responsible use. This may include clearer guidance on when design outputs should be subject to additional review, transparency about tool capabilities and limitations, and engagement between tool developers, researchers, synthesis providers, and biosecurity communities.

The central point is that biosecurity governance should be developed with lower-resource and emerging biotechnology settings, not simply imposed on them. Harmonization will be more credible if shared standards are accompanied by technical assistance, inclusive standard-setting, and capacity-building. This approach helps reduce biosecurity risk while preserving equitable participation in the benefits of synthetic biology.

## 9 Limitations

The four-dimension framework proposed in this review is a conceptual and diagnostic tool, not a validated quantitative model. The dimensions and indicators are intended to support structured governance assessment rather than produce precise risk scores. The framework describes risk as interaction-based, but the relative weight of each dimension will vary across institutional, legal, and technological contexts. It should therefore be used as a structured aid to expert judgement, not as a replacement for context-specific assessment. The framework also does not provide technical screening algorithms, database standards, or implementation protocols, which remain the role of technical bodies, providers, regulators, and dedicated screening research.

The worked case study in Section 7 is a composite illustration rather than an empirical assessment of a specific country. It is designed to show how the framework can be applied across the four dimensions and translated into a tier assignment. Its conclusions should not be interpreted as a risk ranking or governance evaluation of any named jurisdiction. Applying the framework to real settings would require systematic data collection, stakeholder engagement, and jurisdiction-specific legal and technical review.

Several practical limitations also affect measurement. Data on active synthesis providers, benchtop synthesis devices, screening implementation, customer verification practices, and synthesis activity outside voluntary standards are not always publicly available or comparable across jurisdictions. Similarly, assessing governance strength and liability clarity may require detailed legal expertise and access to regulatory information that is not consistently documented. In practice, the framework will often rely on informed estimates and proxy indicators. Future work should refine and test measurement approaches for each dimension to strengthen the framework’s operational utility.

## 10 Conclusion

Synthetic nucleic acid technology continues to expand in capability, accessibility, and geographic distribution. The governance frameworks developed to manage its biosecurity risks have been valuable in addressing threats linked to known pathogenic sequences, but they were designed for a more centralized and less computationally enabled environment than the one that now exists. Sequence screening and customer verification remain essential safeguards and should be retained and strengthened. The argument of this review is not that these measures have failed, but that they are insufficient on their own to address the structural pressures now shaping the field.

These pressures include expanding computational design capability, including AI-assisted and generative tools that may produce sequences harder to assess through homology-based screening alone; broader access to commercial and benchtop synthesis platforms; fragmented governance across jurisdictions with different legal authority and implementation capacity; and persistent gaps in liability and attribution across the design-synthesis-use pathway. Their combined effect is to expose the limits of a governance architecture built mainly around provider-level screening checkpoints.

The four-dimension framework proposed in this review, integrating design capability, access, governance strength, and liability and attribution clarity, provides a structured way to assess how these pressures interact in specific contexts. The four-tier escalation model then links these dimension profiles to proportionate policy responses and assigns responsibilities to synthesis providers, research institutions, governments, funders, and international bodies. These tools are intended to complement existing screening standards and national regulatory frameworks, not replace them.

Proportionality and equity are central to this approach. A governance model that concentrates compliance burdens in lower-resource settings without support, or treats capacity gaps primarily as risk factors rather than governance development needs, is unlikely to produce meaningful harmonization. Biosecurity strengthening should therefore be paired with capacity-building, technical assistance, and inclusive standard-setting so that responsible innovation is supported alongside risk reduction.

The challenges posed by synthetic nucleic acid technology in the AI era will not be resolved by any single framework. This review offers a systems-level diagnostic that can help regulators, providers, research institutions, and international bodies ask more structured questions about where risk is concentrated, what responses are proportionate, and how screening and oversight can evolve as the technological landscape changes.
